# Arginase‐1+ bone marrow myeloid cells are reduced in myeloproliferative neoplasms and correlate with clinical phenotype, fibrosis, and molecular driver

**DOI:** 10.1002/cam4.5542

**Published:** 2022-12-15

**Authors:** Arturo Bonometti, Oscar Borsani, Elisa Rumi, Virginia Valeria Ferretti, Claudia Dioli, Elena Lucato, Marco Paulli, Emanuela Boveri

**Affiliations:** ^1^ Unit of Anatomic Pathology IRCCS San Matteo Foundation Pavia Italy; ^2^ Pathology Unit Humanitas Clinical and Research Center IRCCS Rozzano Italy; ^3^ Department of Molecular Medicine University of Pavia Pavia Italy; ^4^ Division of Hematology Fondazione IRCCS Policlinico San Matteo Pavia Italy; ^5^ Unit of Clinical Epidemiology and Biostatistics Fondazione IRCCS Policlinico San Matteo Pavia Italy

**Keywords:** CALR, immune microenvironment, MDSC, myelofibrosis, myeloproliferative neoplasms

## Abstract

**Introduction:**

Philadelphia‐negative myeloproliferative neoplasms (MPN) are clonal myeloid proliferative disorders characterized by sustained systemic inflammation. Despite its renowned importance, the knowledge concerning the inflammatory pathophysiology of these conditions is currently limited to studies on serum cytokines, while cellular immunity has rarely been investigated.

**Methods:**

In the present study, we targeted Arginase‐1 immunosuppressive myeloid cells in the bone marrow of MPN patients and healthy controls and investigated their clinical and prognostic significance. We demonstrated that MPN are characterized by a significant reduction of bone marrow immunosuppressive cells and that the number of these cells significantly correlates with several clinical and histopathological features of diagnostic and prognostic importance. Moreover, we identified an unreported correlation between a reduction of Arginase‐1+ bone marrow cells and the presence of CALR mutations, linking tumor‐promoting immunity and molecular drivers. Finally, we postulate that the reduction of bone marrow Arginase‐1+ immunosuppressive cells may be due to the migration of these cells to the spleen, where they may exert systemic immunomodulatory function.

**Conclusion:**

Altogether, this study preliminary investigated the contribution of cellular immunity in the pathogenesis of myeloproliferative neoplasms and identified a possible interesting therapeutic target as well as a set of new links that may contribute to unraveling the biological mechanisms behind these interesting hematological neoplasms.

## 1 INTRODUCTION

“Classic” Philadelphia chromosome‐negative myeloproliferative neoplasms (MPN) are a heterogeneous group of clonal disorders of hematopoiesis presenting during the adulthood and characterized by the proliferation of one or more myeloid lineages, bone marrow (BM) hypercellularity with effective cell maturation, frequent peripheral cytoses, and recurrent molecular alterations in *JAK2*, *CALR*, and *MPL* genes. MPN includes polycythemia vera (PV), essential thrombocythemia (ET), and primary myelofibrosis (PMF). All these conditions, and especially PMF, may progress toward a fibrotic involution of BM, frequently associated with cytopenia and splenomegaly and shorter overall survival (OS).^1^

Since the discovery of the *JAK2*V617F mutation, the molecular landscape of MPN has been widely studied.^2^ However, much less is known concerning the contribution of immunity to the pathogenesis and evolution of these conditions.^3^ Furthermore, the specific role of myeloid immunity seems of particular interest, as MPN may be considered tumors of innate immune cells progenitors (e.g., granulocyte‐monocyte lineage).^1^ In recent years, tumor‐associated myeloid cells have been in the spotlight, thanks to the demonstration of their fundamental role in immune‐regulation of the tumor microenvironment, and their predictive and prognostic significance in a wide spectrum of cancers. Probably, the most investigated among these cell types are the myeloid‐derived suppressor cells (MDSCs) which are the major conductors of Th1‐suppression in cancer.^4^ MDSCs exert their function through the production of different soluble mediators (e.g., TGF‐β and IL10), expression of surface molecules (e.g., PDL‐1 and CD40), and depletion of amino acids essential for T‐cell functions (e.g., arginine and tryptophan).^5^ Interestingly, the enzyme necessary for arginine deprivation ‐Arginase‐1‐ is also one of the more specific functional markers of MDSCs. Indeed this protein is only expressed by hepatocytes and MDSCs, in human tissues.^6^, ^7^ Arginase‐1‐mediated arginine deprivation induces immunosuppressive effects on T‐cells through downregulation of CD3ζ, cycle‐cell arrest, inhibition of immune synapse with antigen‐presenting cells, and negative metabolic regulation. Therefore Arginase‐1 represents a promising indicator of immunosuppressive activity in cancer.^6^

In MPN, the pathogenic contribution of immunity has been investigated almost exclusively by analyzing the biological and prognostic significance of serum cytokines and chemokines.^8^ However, almost nothing is known concerning the role of cellular immunity in MPN pathogenesis, and apparently, no study ever examine the presence of immunosuppressive myeloid cells in the BM of MPN patients.^9^

Our aim is to deepener the knowledge concerning the presence of immunomodulatory myeloid cells in the human BM, in controls and MPN patients fostering research on this relevant but under investigated field of immune‐oncology. For this purpose, in this study, we investigated the presence of Arginase‐1+ myeloid cells in the BM of MPN patients and controls and analyzed its correlation with the clinical‐pathological phenotype, molecular driver, and prognosis.

## 2 METHODS

We enrolled a series of consecutive patients who received a diagnosis of MPN at IRCCS San Matteo Foundation, Pavia, Italy, between January 2003 and December 2020 and for whom an adequate formalin‐fixed paraffin‐embedded (FFPE) BM biopsy performed at presentation was available together with complete clinical, genetic, and follow‐up data. Diagnosis of MPN was made following the 2017 World Health Organization criteria.^1^ Mutational status was evaluated on DNA extracted from peripheral blood polymorphonuclear cells. Granulocyte *JAK2* V617F mutation, *CALR* exon 9 mutations, and *MPL* exon 10 mutations were assessed as previously reported.^10^, ^11^, ^12^

We specifically distinguished MPN diagnosis between those with absent/low (grade ≤1) BM fibrosis (i.e., PV, ET, and prefibrotic‐PMF) and those with overt (grade ≥2) BM fibrosis (i.e., overt‐PMF and post‐PV myelofibrosis) to assess the possible correlation between the percentage of Arginase‐1+ BM myeloid cells and BM fibrosis. This last being the main and more prognostically significant post‐inflammatory event in MPN pathogenesis.^13^

We obtained a 3 μm‐thick section from the FFPE BM biopsy block of each patient and stained it with the immunohistochemical antibody against Arginase‐1 (clone A‐2 by Santa Cruz Biotechnology) using the DAKO Omnis automated immunostainer (DAKO Cytomation). Moreover, given the absence of a description of the presence of Arginase‐1‐positive cells in human BM, we also gather the BM biopsies of 12 healthy sex‐ and age‐matched controls (i.e., negative staging biopsy from untreated, follicular lymphoma patients with clinical stage I or II) and stained them with the same antibody. For each stained BM slide, the percentage of Arginase‐1‐positive cells over the BM cellularity was assessed by three experienced hematopathologists (AB, EB, MP). A cut‐off of 20% was set according to a preliminary ROC analysis (data not shown). MPN patients were thus divided into two groups accordingly that is with ≤20% or >20% of Arginase‐1‐positive BM cells.

### 2.1 Statistical analysis

Categorical variables were described as counts and percentage. Quantitative variables were summarized as median and interquartile range (IQR). Association between categorical variables was tested via Fisher's exact test. The comparison of quantitative variables between two groups of patients was evaluated by Wilcoxon test for unpaired data. Reverse Kaplan–Meier method was implemented to estimate length of follow‐up.

Overall Survival (OS) was calculated as the time between diagnosis (left‐truncated for date of BM biopsy) and death or last follow‐up, and was estimated by Kaplan–Meier product‐limit method. Proportional hazard Cox models were carried out in order to adjust the effect of Arg1 on OS for the effect of confounders. The cumulative incidence of leukemic evolution was calculated as the time between diagnosis (left‐truncated for date of BM biopsy) and leukemic evolution or death/last follow‐up and was estimated in a competing risk approach, considering death without evolution as a competing event. The Fine&Gray model was used to evaluate the effect of Arg1 on the cumulative incidence of evolution. Due to the low number of evolutions, only a univariable model was carried out.

Two‐sided *p*‐values <0.05 were considered statistically significant.

All statistical analyses were performed using Stata 17 (StataCorp. 2021. Stata Statistical Software: Release 17. StataCorp LLC.).

## 3 RESULTS

### 3.1 Arginase‐1 staining in MPN patients and healthy controls

**FIGURE 1:**
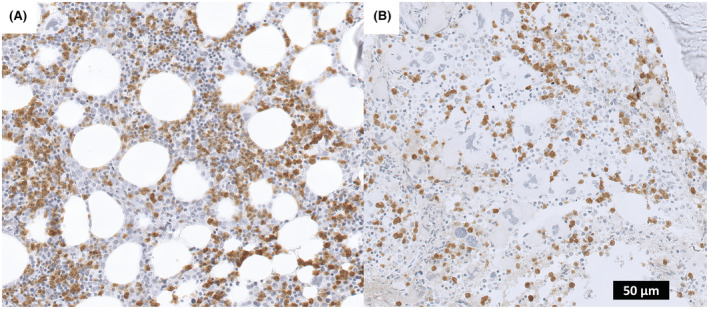
Immunohistochemical staining against Arginase‐1 (100× magnification) in a PV patient with >20% of positive cells (A), and a prefibrotic PMF patient with <20% of positive cells (B).

Overall, we enrolled 12 healthy controls and 128 MPN patients. Both in MPN patients and controls, Arginase‐1 stained medium‐sized cells with single large un‐lobated or folded nuclei and moderate cytoplasm. These cells were located mainly in para‐trabecular areas or at the center of the BM interstitium adjacent to arterioles. On a morphological and architectural ground, these cells were suggestive of myeloid precursors (e.g., promyelocytes and myelocytes) and to a lesser extent for band cells and mature granulocytes. Nevertheless, the percentage of Arginase‐1+ BM cells (%Arg1) did not match the total number of BM myeloid cells, as evaluated morphologically and with MPO immunostaining. Moreover, in both controls and patients, erythroid precursors, megakaryocytes, lymphocytes, and mast cells, resulted Arginase‐1‐negative (Figure 1).

**FIGURE 2:**
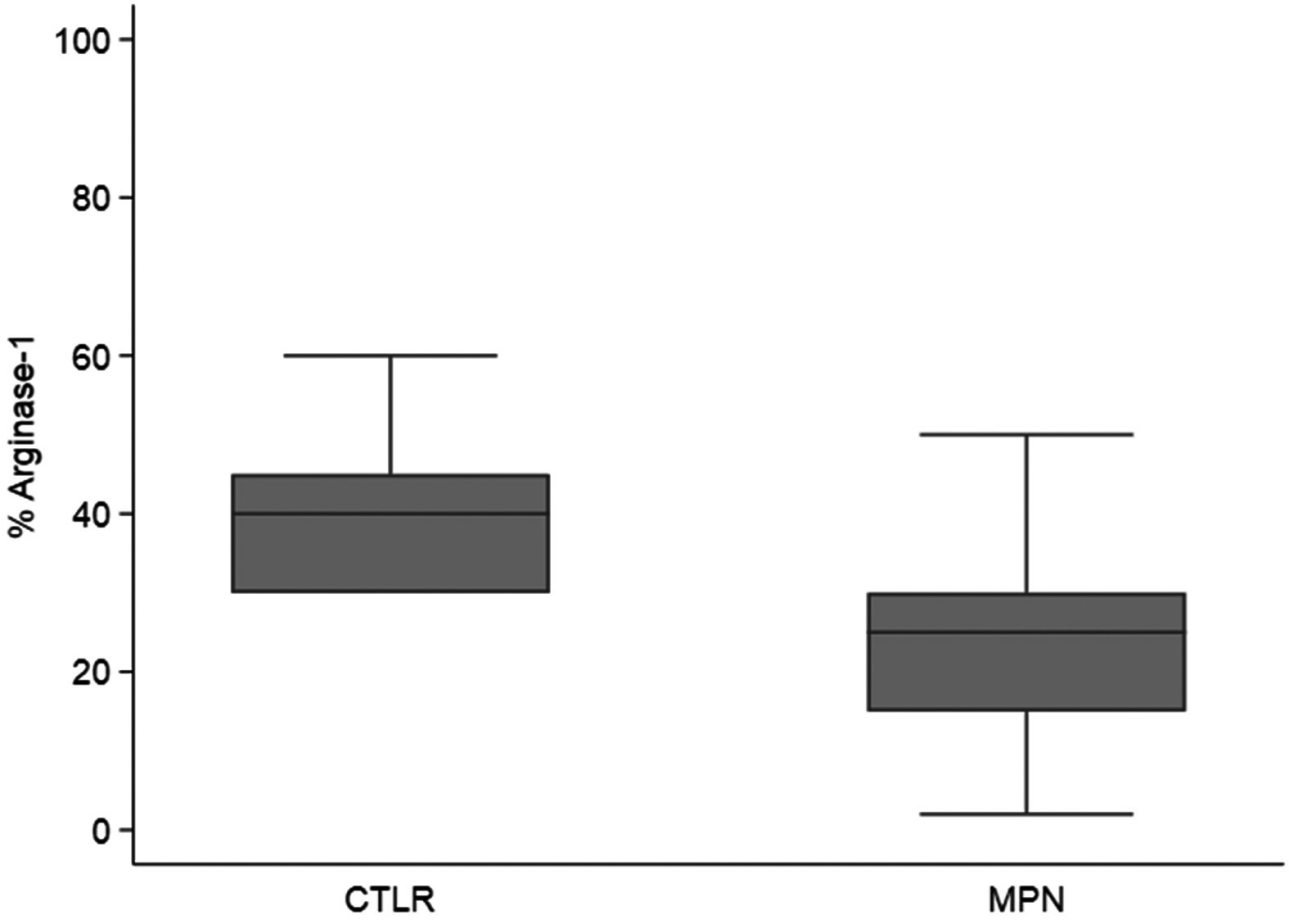
Comparison of percentage of Arginase‐1+ bone marrow cells (%Arg1) between MPN patients and healthy controls (CTRL) (*p* < 0.001).

Arginase‐1 marked a median of 40% BM cellularity (IQR: 30%–45%), in healthy controls, compared to a median of 25% in the BM cellularity (IQR: 15%–30%) in MPN patients (*p* < 0.001, Figure 2).

Interestingly we observed a reduction of the median %Arg1 moving from controls (40%) to PV (30%), ET (25%), prefibrotic PMF (25%), post‐PV myelofibrosis (20%), and overt PMF (18%). In other words, the %Arg1 was reduced in MPN compared to controls and in those MPN diagnostic categories characterized by a BM fibrosis of grade ≥2 –that is overt PMF and post‐PV myelofibrosis‐ (*p* < 0.001, Table 1).

**TABLE 1:** Clinical, histopathological, and molecular features of our patients' cohort divided according to the percentage of bone marrow Arginase‐1+ cells above or below 20%

	Total	BM ARG1 ≤ 20% (*n* = 60)	BM ARG1 > 20% (*n* = 68)	*p*‐value
Age, median (IQR)	63 (51–71)	65 (53–71)	62 (49–71)	0.352
Gender, *n* (%)				0.723
Males	71 (55.5%)	32 (53.3%)	39 (57.4%)	
Females	57 (44.5%)	28 (46.7%)	29 (42.6%)	
MPN diagnosis, *n* (%)				<0.001
BM fibrosis ≤1	82 (64.1%)	28 (46.7%)	54 (79.4%)	
BM fibrosis ≥2	46 (35.9%)	32 (53.3%)	14 (20.6%)	
HCT (%), median (IQR)	42.5 (37.6–48.1)	39.4 (32.9–46.5)	45.0 (41.2–50.9)	<0.001
Hb (g/dl), median (IQR)	13.3 (11.4–15.3)	12.4 (9.9–14.3)	14.1 (12.6–15.9)	<0.001
WBC × 10^3^/mmc, median (IQR)	9.9 (7.6–13.0)	8.3 (6.8–12.6)	10.4 (8.5–13.6)	0.008
PLT × 10^3^/mmc, median (IQR)	524 (264–730)	503 (230–730)	552 (363–733)	0.258
sCD34+ cells/μl, median (IQR)	8 (4.1–55.0)	14.0 (4.4–63.6)	6.8 (3.5–55.0)	0.386
LDH (U/L), median (IQR)	280 (219–435)	369 (250–550)	249 (200–331)	0.004
EPO (mU/ml), median (IQR)	7.4 (3.7–19.6)	12.6 (4.7–33.0)	4.6 (3.4–9.6)	0.003
Splenomegaly, *n* (%)	53 (41.4%)	30 (50.0%)	23 (33.8%)	0.074
Splenomegaly (cm), median (IQR)	0 (0–3)	0.5 (0.0–5.5)	0.0 (0.0–1.5)	0.021
Hepatomegaly, *n* (%)	27 (21.1%)	13 (21.7%)	14 (20.6%)	>0.90
Hepatomegaly (cm), median (IQR)	0 (0–0)	0 (0–0)	0 (0–0)	>0.90
Mutational status, *n* (%)				0.003
JAK2 V617F	100 (78.1%)	40 (66.7%)	60 (88.2%)	
CALR	26 (20.3%)	19 (31.7%)	7 (10.3%)	
Exon 12	1 (0.8%)	0 (0.0%)	1 (1.5%)	
Triple negative	1 (0.8%)	1 (1.7%)	0 (0.0%)	

### 3.2 Arginase‐1 and MPN patients' clinical and molecular features

MPN patients included 28 PV, 26 ET, 28 prefibrotic‐PMF, 32 overt‐PMF, and 14 post‐PV myelofibrosis with a total of 71 males and 57 females and a median age at diagnosis of 62 years (patients' clinical features are summarized in Table 1). A hundred patients (78%) were *JAK2*V617F‐mutated, with a median allele frequency of 43% (IQR: 19–65), while 26 (20%) were mutated for *CALR*, including 20 with type‐1 and six with type‐2 mutations. A single PV patient displayed an exon‐12 *JAK2* mutation and the last patient was triple‐negative. No patient showed mutations in MPL gene. The median follow‐up from diagnosis was 64 months (IQR: 38–161 months), during which we observed 36 deaths (for both disease‐related and unrelated causes) and 12 leukemic evolutions. Compared to patients with >20%Arg1, those with ≤20%Arg1 include patients with higher serum EPO levels (12.6 vs. 14.6 mU/ml), lower hematocrit (39% vs. 44%), lower serum hemoglobin (12.4 g/dl vs. 14.1 g/dl) and thus a higher number of patients with anemia (44% vs. 19%). Furthermore, they also display a higher median serum CD34+ cells (14/mcL vs. 6.8/mcL) and LDH level (369 U/L vs. 249 U/L), and include a higher number of patients with BM fibrosis grade ≥2 (53% vs. 21%), splenomegaly (50% vs. 34%) and *CALR* mutations (32% vs. 10%). The two groups showed similar JAK2V617F allele frequencies in JAK2‐mutated patients (46% vs. 38% *p* = 0.151).

### 3.3 Arginase‐1 and MPN patients' outcome

**FIGURE 3:**
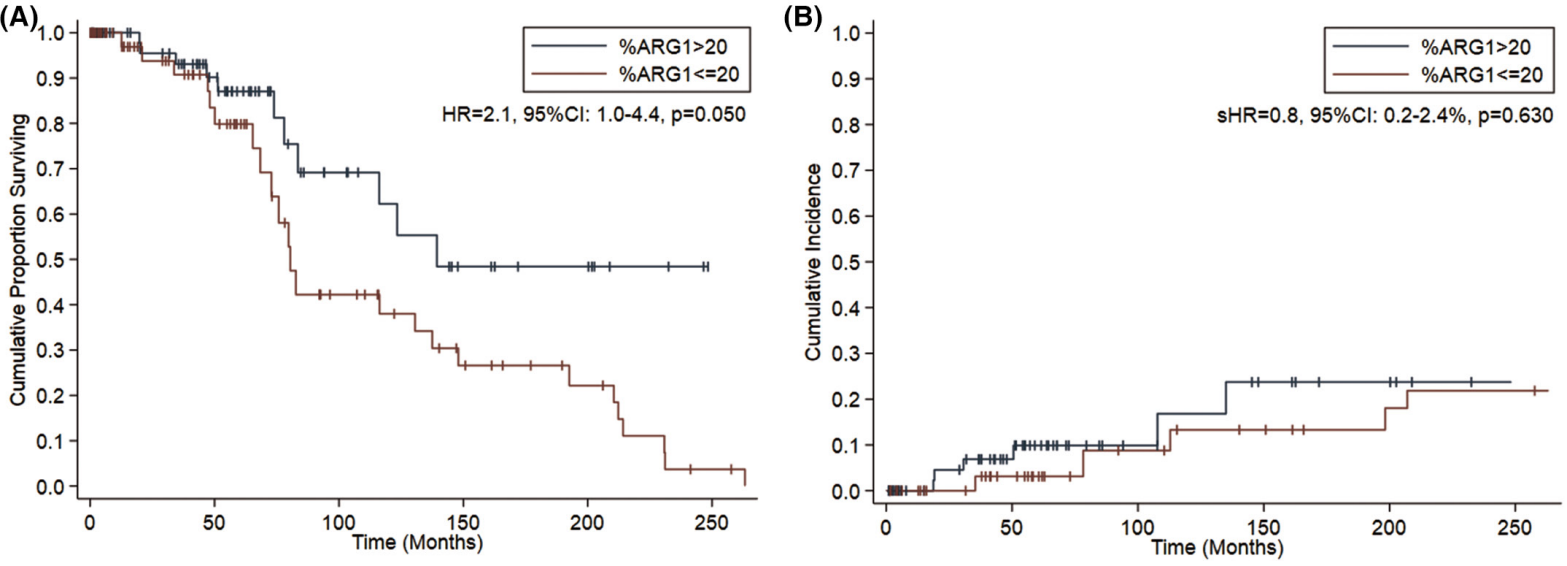
Overall survival (A) and Cumulative Incidence of leukemic evolution (B) according to the percentage of Arginase‐1+ cells in myeloproliferative neoplasms patients

In univariable analysis, overall survival was shorter in patients with Arginase‐1 < 20% respect to those with Arginase‐1 > 20% (60‐months OS: 79.8%, 95%CI: 60.4–90.5%, vs. 87.1%, 95%CI: 71.4%–94.5%, respectively; HR = 2.1, 95%CI: 1.0–4.4, *p* = 0.050; Figure 3A). Nevertheless, this trend was not confirmed both after adjusting for age (HR = 1.7, 95%CI: 0.7–3.7, *p* = 0.223) and after adjusting for BM fibrosis grade ≥2 (HR = 1.7, 95%CI: 0.8–3.7, *p* = 0.172).

Furthermore, the cumulative incidence of leukemic evolution was overlapping between Arginase‐1 < 20% and Arginase‐1 > 20% (60‐months cumulative incidence: 3.1%, 95%CI: 0.2%–13.7% and 9.9%, 95%CI: 3.1–21.4%, respectively; sHR = 0.8, 95%CI: 0.2–2.4%, *p* = 0.630; Figure 3B).

## 4 DISCUSSION

Cancer may hijack normal hematopoiesis, leading to expansion of immature myelo‐monocytic cells with immunosuppressive activity (e.g., MDSCs). This process is similar to emergency hematopoiesis associated to systemic inflammatory states.^14^, ^15^, ^16^, ^17^ MDSCs are immunoregulatory cells that contribute to dampening the antitumor immunity and favor the genesis of a tumor‐promoting local and systemic environment, thus also representing interesting therapy targets.^6^, ^18^, ^19^ MDSCs may act through cytokine production, immunosuppressive cells induction or direct Th1‐cells inhibition.^5^ Moreover, recent studies indicates the spleen as the major site of MDSCs‐mediated T‐cell suppression, and a strategic site of cancer‐related immune‐modulation.^20^, ^21^, ^22^

The main issue studying MDSCs concerns their identification. According to the last consensus, they may be identified only by the demonstration of both positivity to a specific marker (e.g., CD11b, CD33, CCR2, CXCR4, Arginase‐1, and pSTAT3) and of immunosuppressive functions.^7^, ^23^ Nevertheless, the immunohistochemical positivity to Arginase‐1, allows for meeting both needs at once. Indeed, it is only expressed by MDSCs within human blood cells and also attests to the immunosuppressive function of positive cells.^6^, ^7^

As “a human inflammation model for cancer development” MPN are characterized by increase of serum inflammatory mediators (e.g., CRP, IL‐6, IL‐8, TNF‐α, and Th2 cytokines), clinical similarities with systemic inflammatory conditions (e.g., fatigue, fever, vasomotor symptoms, thrombocytosis, anemia, and spleen enlargement) and post‐inflammatory stigmata such as BM fibrosis.^3^, ^8^, ^9^ Indeed, many effective treatments for MPN also display an anti‐inflammatory effect.^3^ Despite this, data on the role of cellular immunity in MPN are lacking. A single study investigated the role of MDSCs (identified as Lin‐, CD11b+, CD14‐, CD33+ cells, capable of T‐cells suppression) in the serum of a small series of MPN patients. Compared with healthy controls, MDSCs and Arginase‐1 mRNA were significantly increased in MPN patients' serum, without differences among diagnostic categories or demonstration of any correlation with the clinical and molecular features of the patients.^24^ More recently, Molitor et al. studied the presence of CD68+ or CD163+ macrophages in the BM of MPN patients, highlighting a correlation between their percentage and diagnostic category.^25^

Bearing in mind the model of emergency myelopoiesis and the BM origin of MDSCs, we investigate the presence of Arginase‐1+ cells in BM biopsies of healthy controls and MPN patients. For MPN we included five different diagnostic groups according to WHO classification and the grade of BM fibrosis.^1^ As fibrosis is a major post‐inflammatory complication in MPN, we thought it to represent an important variable to consider independently, thus we had taken post‐PV myelofibrosis and overt‐PMF patients apart from PV and prefibrotic PMF ones in our study and statistical analysis.

Our data documented for the first time the presence of cells expressing immunomodulatory molecules in BM tissue samples of healthy controls, as well as a significant reduction of %Arg1 in MPN patients. Moreover, we highlighted the presence of correlations between %Arg1 and multiple clinical‐pathological and molecular features in MPN patients. The statistical significance of these correlations suggests a contribution of Arginase‐1+ BM cells in the generation of diagnostic and prognostic features that were not previously recognized or reported such as anemia, BM fibrosis, and splenomegaly.

Interestingly, the literature concerning MDSCs biology seems to support our findings and allowed us to further deduce missing links between MDSCs, BM Arginase‐1+ cells, and the clinical‐pathological phenotype of our patients. As an example, MDSCs are capable to induce anemia, through their production of TNF‐α: the main cytokine regulator of erythropoiesis.^26^, ^27^ Even more interestingly, MDSCs may stimulate fibrosis either by secreting pro‐fibrotic mediators (IL6, TGF‐β, VEGF e PDGF), by differentiating in collagen‐producing fibrocytes, or by releasing proline (a major component of collagen and catabolite of Arginase‐1‐mediated reactions).^28^, ^29^ Furthermore, the profibrotic function of MDSCs have already been described in different organs and conditions.^30^, ^31^

In addition, MDSCs are capable to migrate from the peripheral blood toward the spleen, and hereby expanding, thanks to their expression of CCR2 and CXCR4 and hence their ability to respond to CXCL12 and CCL2 gradients produced by splenic endothelia in emergency myelopoiesis or MPN.^20^, ^22^ Interestingly, we recently demonstrated the splenic expansion of MDSC in MPN patients.^32^

Eventually, our analysis also highlighted a correlation between *CALR* mutation and reduction of %Arg1, linking immune‐modulation and molecular drivers in MPN. Interestingly a serum and splenic expansion of MDSCs has been recently described in *CALR*‐mutant MPN mouse models and listed as a possible cause of the suppression of mutant *CALR*‐specific T‐cells in *CALR*‐mutated MPN.^33^, ^34^, ^35^

Finally, the reduction of %Arg1 in MPN patients also correlated with a shorter OS in univariable analysis, even though significativity was lost in age‐ and BM fibrosis‐adjusted analyses.

All in all, the available data together with our results, suggest a possible BM reduction of *bona‐fide* MDSCs as a result of their migration toward the peripheral blood and spleen. In this last organ, they may exert well‐described immunomodulatory functions on T‐cells and secrete soluble mediators, including profibrotic cytokines and growth factors thus regulating inflammation and its effects at a systemic level and influencing clinical phenotype and patients' prognosis. Our data further validate the importance of inflammation and immunomodulation in the pathogenesis and progression of MPN and contributes to unraveling the role of cellular immunity in this scenario, further suggesting MDSCs as a possible therapeutic target in MPN. However, future studies should scrutinize the biological mechanisms underlying each correlation that emerged from this paper.

## AUTHOR CONTRIBUTIONS

**Arturo Bonometti:** Conceptualization (lead); data curation (lead); formal analysis (supporting); funding acquisition (supporting); investigation (lead); methodology (lead); project administration (lead); resources (equal); software (equal); supervision (equal); validation (equal); visualization (equal); writing – original draft (lead); writing – review and editing (lead). **Oscar Borsani:** Conceptualization (supporting); data curation (equal); project administration (equal); writing – original draft (equal); writing – review and editing (equal). **Elisa Rumi:** Conceptualization (equal); data curation (equal); formal analysis (equal); investigation (equal); project administration (equal); writing – original draft (equal); writing – review and editing (equal). **Virginia V. Ferretti:** Conceptualization (equal); formal analysis (lead); methodology (equal); project administration (equal); software (equal); writing – original draft (equal); writing – review and editing (equal). **Claudia Dioli:** Methodology (equal). **Elena Lucato:** Methodology (equal). **Marco Paulli:** Supervision (supporting). **Emanuela Boveri:** Conceptualization (equal); data curation (equal); investigation (equal); methodology (equal); project administration (equal); supervision (equal); writing – original draft (equal); writing – review and editing (equal).

## FUNDING INFORMATION

The study was supported by grants from the Italian Ministry of Health for young researchers (GR‐2016‐02361272) to ER, by Associazione Italiana per la Ricerca sul Cancro (AIRC, Milan, Italy) (IG 2021 ID 25703) to ER, and by Associazione Italiana per la Ricerca sul Cancro (AIRC, Milan, Italy) through the project “Actionable targets in clonal progression and systemic spreading of myeloid neoplasms”, MYNERVA project (Project Code: 21267) to ER.

## CONFLICT OF INTEREST

The authors have no competing financial interests.

## ETHICS APPROVAL STATEMENT

The study was approved by the ethics committee of the IRCCS San Matteo Foundation of Pavia.

## PATIENT CONSENT STATEMENT

Samples and clinical data were obtained with patients' written informed consent.

## CLINICAL TRIAL REGISTRATION

N/A.

## Data Availability

All data of the study are available upon request.
